# PD-L1 Tumor Expression as a Predictive Biomarker of Immune Checkpoint Inhibitors’ Response and Survival in Advanced Melanoma Patients in Brazil

**DOI:** 10.3390/diagnostics13061041

**Published:** 2023-03-09

**Authors:** Bruna Pereira Sorroche, Renan de Jesus Teixeira, Caio Augusto Dantas Pereira, Iara Viana Vidigal Santana, Lazar Vujanovic, Vinicius de Lima Vazquez, Lidia Maria Rebolho Batista Arantes

**Affiliations:** 1 Molecular Oncology Research Center, Barretos Cancer Hospital, Barretos 14784-400, Brazil; brusorroche@hotmail.com (B.P.S.);; 2 Department of Clinical Oncology, Barretos Cancer Hospital, Barretos 14784-400, Brazil; 3 Department of Pathology, Barretos Cancer Hospital, Barretos 14784-400, Brazil; 4 UPMC Hillman Cancer Center, University of Pittsburgh, Pittsburgh, PA 15260, USA; 5 Department of Otolaryngology, University of Pittsburgh, Pittsburgh, PA 15260, USA; 6 Melanoma, Sarcoma, and Mesenchymal Tumors Surgery Department, Barretos Cancer Hospital, Barretos 14784-400, Brazil

**Keywords:** melanoma, immune checkpoint blockade, response, PD-L1 expression

## Abstract

Immune checkpoint blockade (ICB) agents are prominent immunotherapies for the treatment of advanced melanoma. However, they fail to promote any durable clinical benefit in a large cohort of patients. This study assessed clinical and molecular predictors of ICB response and survival in advanced melanoma. A retrospective analysis was performed on 210 patients treated with PD-1 or CTLA-4 inhibitors at Barretos Cancer Hospital, Brazil. PD-L1 expression was assessed by immunohistochemistry using formalin-fixed paraffin-embedded tumor tissues collected prior to ICB therapy. Patients were divided into responders (complete and partial response and stable disease for more than 6 months) and non-responders (stable disease for less than 6 months and progressive disease). Among them, about 82% underwent anti-PD-1 immunotherapy, and 60.5% progressed after the ICB treatment. Patients that received ICB as first-line therapy showed higher response rates than previously treated patients. Higher response rates were further associated with superficial spreading melanomas and positive PD-L1 expression (>1%). Likewise, PD-L1 positive expression and *BRAF* V600 mutations were associated with a higher overall survival after ICB therapy. Since ICBs are expensive therapies, evaluation of PD-L1 tumor expression in melanoma patients should be routinely assessed to select patients that are most likely to respond.

## 1. Introduction

Cancer is considered a worldwide public health concern that affects a significant and growing number of individuals, especially among developing countries [1]. The recent success of immunomodulatory agents in patients with refractory solid tumors demonstrates that activating the immune system is effective as a therapeutic modality [2]. However, tumor cells develop mechanisms to evade recognition and activation of the immune response in a dynamic process called immunoediting [2].

Melanoma is the most aggressive type of skin cancer [3,4], responsible for 325,000 new cases and 57,000 deaths worldwide in 2020 [5]. The treatment for advanced melanomas may involve complex surgery, radiation therapy, and various systemic therapeutic approaches [6]. When the tumor is diagnosed at stage III, the 5-year overall survival rate is 66.5%, while for cases at stage IV, it is around 25.0% [7]. In the last decade, the use of immune checkpoint blockade (ICB) agents has significantly improved melanoma patients’ survival outcomes [8,9,10]. Nevertheless, about 30% of these patients benefit from the treatment as monotherapy [11,12], and the cellular and molecular aspects associated with this response need to be further elucidated [13].

Previous studies have identified an increased expression of programmed death-ligand 1 (PD-L1) on tumor cells as the most common biomarker to predict response to ICB [14,15]. However, the use of this marker is controversial due to the overall survival benefit of ICB regardless of PD-L1 tumor expression levels [16,17].

As in the wider world, ICB has been gaining ground in Brazil [18]. Thus, this study aimed to investigate the PD-L1 tumor expression profile as a predictive biomarker of response to ICB therapy in Brazilian patients with advanced melanoma in a real-world scenario. Here, we demonstrated that the use of PD-L1, together with clinical information, could be implemented in the clinical setting to identify patients that might benefit from ICB treatments.

## 2. Materials and Methods

### 2.1. Patients

We selected 210 consecutive patients with advanced melanoma who underwent immunotherapy treatment with antibodies against programmed cell death protein 1(PD-1) and/or cytotoxic T-lymphocyte-associated protein 4 (CTLA-4) at Barretos Cancer Hospital (BCH) between January 2011 and December 2021.

Epidemiological, clinical, and anatomopathological data were collected from medical records and internal data management systems. Clinical response was assessed by clinical oncologists based on the routine radiological evaluation. Patients were subjected to positron emission tomography with computed tomography (PET/CT) every 3 months for disease follow-up and treatment response evaluation. In addition, when brain metastasis was suspected, the patients underwent magnetic resonance imaging (MRI). Inclusion criteria included every advanced melanoma patient with irresectable (stage III) or metastatic (stage IV) disease treated with ICB, with biological tumor samples available. Patients without measurable disease, treated with ICB in the adjuvant setting, were excluded from this study. 

### 2.2. PD-L1 Tumor Expression

The most representative areas of the formalin-fixed paraffin-embedded (FFPE) tumor tissues were selected for PD-L1 expression analysis by immunohistochemistry (IHC). Samples from all patients were collected prior to ICB treatment. The reaction was performed using the Benchmark^®^ ULTRA platform using the rabbit anti-PD-L1 monoclonal antibody (clone E1L3N, 1:200 dilution, Cell Signaling Technology). The Optiview DAB visualization system was used for PD-L1 protein detection in 5 μm sections, according to the manufacturer’s specifications. A minimum of 100 viable tumor cells on the slide was required for an adequate evaluation of PD-L1. 

PD-L1 expression was determined by a specialized pathologist using two scores: the tumor proportion score (TPS) and the combined positive score (CPS). The TPS evaluates the percentage of viable tumor cells that show partial or complete membrane staining at any intensity. The CPS evaluates the total number of PD-L1-positive stained cells (tumor and immune system cells) among the total number of viable tumor cells on the slide. Both indexes range from 0 to 100% and were considered positive when greater than or equal to 1%. 

### 2.3. BRAF and NRAS Mutation Status

BRAF and NRAS mutation status were evaluated by next-generation sequencing using the TruSight Tumor 15 panel on MiSeq instrument (Illumina, San Diego, CA, USA), as previously described [19]. When DNA quality did not allow reliable sequencing results, BRAF mutation was evaluated by real-time PCR using the Cobas4800 BRAF V600 Mutation Test assay in the Cobas4800 system (Roche Molecular Diagnostics, Switzerland), following the manufacturer’s instructions.

### 2.4. Statistical Analysis

The statistical software SPSS 23.0 was used for data entry and statistical analysis. Patients with partial (PR), complete response (CR), and stable disease (SD) for more than 6 months were considered as responders to ICB treatment, whereas patients with progressive (PD) and SD for less than 6 months were considered non-responders. Clinical and molecular predictors of ICB response and survival in advanced melanoma were investigated. Survival rates were assessed using the Kaplan–Meier method, and curves were compared using the log-rank test. Multivariate analyses were performed by the logistic regression method, with treatment response as the dependent variable. *p*-values ≤ 0.05 were considered significant. 

## 3. Results

### 3.1. Clinicopathological Data

About 59% of the patients were male and 86.2% were Caucasian. The median age at diagnosis was 53 (ranging from 19–91) years old. The main primary tumor site was the lower limbs (31.4%) followed by the trunk (21.4%). Most patients (81.9%) were treated with anti-PD-1 agents in monotherapy, and 60.5% of them showed disease progression after ICB treatment. Patients were divided into two groups, responders *vs.* non-responders, comprising 83 and 127 patients, respectively. Additional patient information is presented in Table 1. 

**Figure 1:**
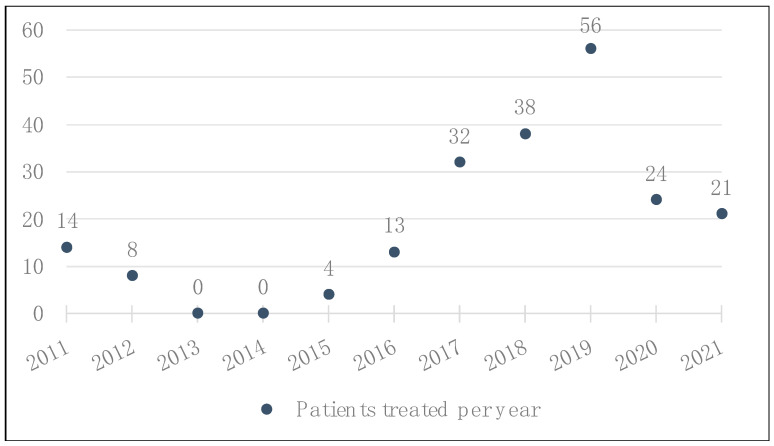
Total number of patients with advanced or metastatic melanoma who received treatment with ICB at BCH, by year.

We observed an increase in indications for ICB at BCH from 2014 until 2019 (Figure 1). However, there was a drop in the number of patients starting treatment with ICB in 2020 due to the impact of the SARS-CoV-2 pandemic on the Brazilian National Health System [20], which made access to this high-cost medication more challenging.

### 3.2. PD-L1 Expression Analysis

All 210 patients were subjected to PD-L1 expression analysis. The source of samples depended on the availability of FFPE blocks in the Department of Pathology. Approximately fifty-four percent of the patients had samples from their primary tumor, while for the remaining patients, only metastatic tumor tissue samples were available (Table 2).

**Figure 2:**
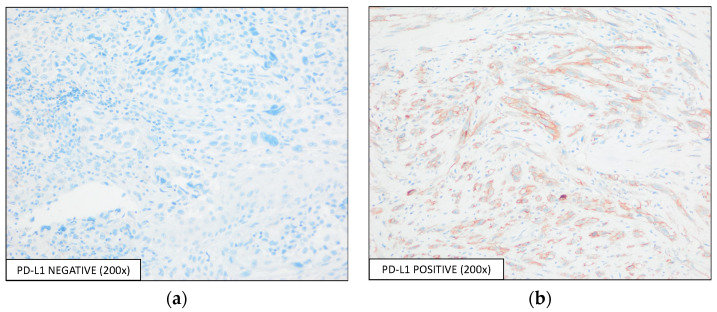
Detection of PD-L1 expression by IHC using the E1L3N antibody, with an optical magnification of 200×. Representative images of PD-L1-(**a**) negative and -(**b**) positive expressions are shown.

**Figure 3:**
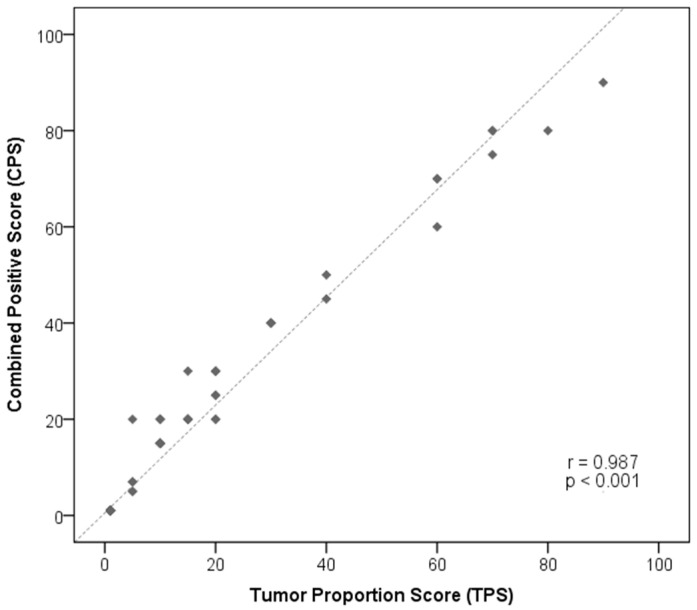
Pearson correlation analysis between TPS and CPS scores to evaluate PD-L1 expression in advanced melanoma patients’ samples.

The evaluation of PD-L1 expression was performed using two scores, TPS and CPS: 44 (21.0%) samples were considered positive by both scoring methods, while 166 (79.0%) samples were considered negative for PD-L1 expression (IHC represented by Figure 2), with a positive correlation between the two scores (Pearson correlation = 0.987, *p*< 0.001, Figure 3).

**Figure 4:**
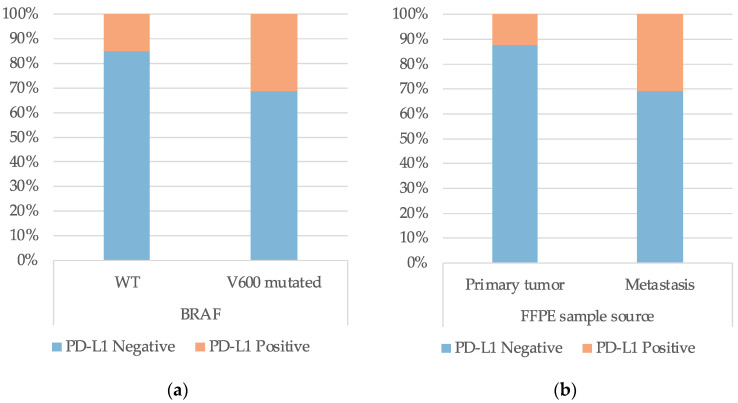
Association of (**a**) *BRAF* V600 mutation status and (**b**) source of FFPE tumor samples with PD-L1 expression profile.

The PD-L1 expression was associated with BRAF V600 mutation status, where 31.2% (24/77) of the patients with mutations in this gene presented PD-L1 positive samples, compared to 15.1% (16/106) of BRAF WT tumors (*p* = 0.011, Figure 4a). PD-L1 expression was also associated with the FFPE sample source, where positive PD-L1 expression was detected in 30.9% (30/97) of the metastatic samples, against 12.4% (14/113) positivity in the primary tumor samples (*p* = 0.001, Figure 4b). The other clinical characteristics evaluated were not significantly associated with the PD-L1 expression profile (data not shown).

### 3.3. Biomarkers of Therapeutical Response and Survival

The clinical characteristics of the patients and the molecular characteristics of the tumor samples were evaluated as predictors of response to ICB treatment. We found that PD-L1 expression lower than 1% in tumor and immune cells, a line of immunotherapeutic treatment other than the first line, an acral lentiginous histological melanoma subtype, and an anti-CTLA-4 treatment as monotherapy were associated with poor responses (Table 1). 

Moreover, the variables of *BRAF* mutation status, ICB treatment agent, ICB treatment line, and PD-L1 expression were included in the multivariate analysis. PD-L1 expression and ICB treatment line remained significant in the model (Table 3). Regarding PD-L1 expression analysis, 85.8% (109/127) of the non-responder patients presented less than 1% of PD-L1 tumor and immune cells’ expression, whereas 31.3% (26/83) of the responder patients presented higher expression (>1%) of this protein with both TPS and CPS methods (*p* = 0.005). In addition, patients treated with ICB therapy as the first line of systemic treatment had higher response rates when compared with patients treated in subsequent lines (49.6% vs. 34.3% in the second line and 13.3% in the third line; *p* = 0.001). 

**Figure 5:**
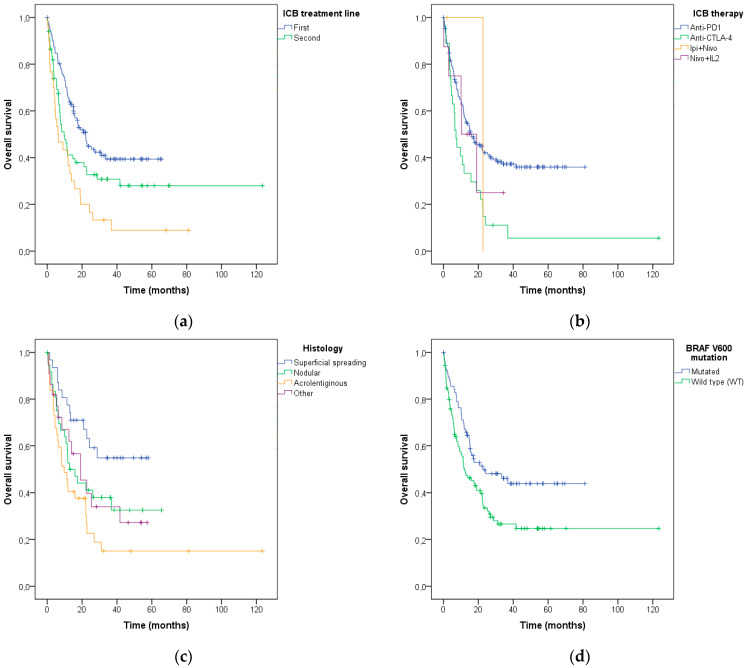

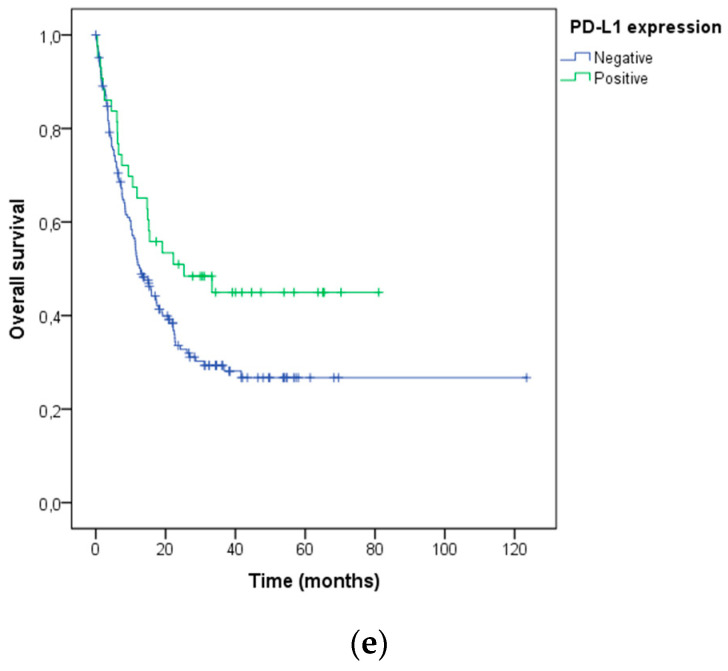
Association of (**a**) systemic ICB treatment line, (**b**) type of ICB, (**c**) histological melanoma subtype, (**d**) *BRAF* V600 mutation status, and (**e**) PD-L1 expression with overall survival after the ICB treatment in advanced melanoma patients.

Kaplan–Meier survival curves were used to estimate the overall survival of the patients after ICB treatment initiation, according to the characteristics described above. Lower survival was associated with the line of the ICB treatment (log-rank *p*< 0.001; Figure 5a), the type of ICB used (log-rank *p* = 0.013; Figure 5b), the histological melanoma subtype (log-rank *p* = 0.009; Figure 5c), *BRAF* V600 mutation status (log-rank *p* = 0.009; Figure 5d) and PD-L1 expression (log-rank *p* = 0.049; Figure 5e). 

Since melanoma patients are treated with ICB therapies in the metastatic setting, we performed the same association analyses using only the metastatic samples (*n* = 97). The ICB treatment line and PD-L1 expression were also associated with the patient’s treatment response (*p* = 0.03 and *p* = 0.004, respectively) and overall survival (*p* = 0.153 and *p* = 0.026, respectively), showing that irrespective of the tumor sample type, PD-L1 expression maintains its relevance.

## 4. Discussion

This study aimed to characterize the clinical and molecular profile of advanced melanoma patients in a Brazilian population and to identify predictive biomarkers of response to ICB therapies. We demonstrated that PD-L1 expression by either TPS or CPS, the line and agent of immunotherapeutic treatment, the histological melanoma subtype, and *BRAF* V600 mutation status were associated with response and/or overall survival after ICB treatment.

Our immune system uses checkpoint receptors to regulate the duration and extent of immune responses to prevent damage to healthy tissues [21]. To avoid being killed by leukocytes, tumor cells exploit this immunosuppressive mechanism by upregulating checkpoint ligands that engage corresponding receptors expressed on immune cells [22]. An example of the mechanism of immune evasion is the ligation of PD-1 expressed on immune cells to PD-L1 expressed on tumor and tumor-infiltrating immune cells, which leads to effector cell inactivation. Therefore, the blockade of immune checkpoints is an approach through which anti-tumor immunity can be re-activated in the tumor microenvironment [21,22]. These agents have been responsible for the induction of long-lasting objective responses in about 40% of melanoma patients, and for the increase in overall survival in advanced-stage melanoma patients [12,23], as well as other tumor types such as non-small cell lung cancer [24], renal cancer [25] and head and neck squamous cell carcinoma [26]. Despite the higher response rates to ICB in comparison with chemotherapy, a large cohort of patients are refractory or acquire resistance to these therapies. Characterization of ICB resistance mechanisms would allow for superior selection of patients that would benefit from these therapies, and for the generation of novel ICB combinations that would benefit a wider population of patients. These developments would lead to more cost- and therapeutically effective ICB strategies [13], which is especially important in Brazil, where most of the healthcare system is public. 

There is a growing need for viable biomarkers to be used in the day-to-day life of an oncologic center, wherein the cost must be feasible for expansion to the entire population. Ayers et al. analyzed the expression of a panel of genes using tumor cells from patients treated with pembrolizumab, and identified signatures related to the immune system which correlated to the clinical benefit of the treatment [13]. Another study demonstrated that global mutational load, neoantigen load, and expression of cytolytic markers in the tumor microenvironment were significantly associated with response in patients with advanced melanoma treated with ipilimumab [27]. Goodman et al. found that tumors with a high mutational load, such as melanoma, are more likely to respond to treatment [28]. Additionally, body mass index (BMI) can be used as a predictor of response to immunotherapy, where obese patients with advanced melanoma respond better, showing greater overall and progression-free survival after treatment with ICBs when compared to non-obese patients [29,30,31]. Unfortunately, all of the aforementioned, except for BMI, are limited by cost for the entire population.

In clinical trials, PD-L1 tumor expression is considered a poor predictor of objective response to checkpoint inhibition, where patients benefit from the treatment independently of PD-L1 expression levels detected in tumor samples [12,23,32]. This can be explained by its dynamic and heterogeneous expression [33,34], which varies upon disease progression [35] and different treatment protocols, including ICB [36]. Moreover, there are several reasons that could explain the heterogeneity in levels of PD-L1 expression as a predictable biomarker of response: differences in tissue samples, PD-L1 expression cut-off, the use of TPS or CPS, as well as the detection technique used. In this study, 34.3% of patients with negative PD-L1 expression presented objective responses after the ICB treatment, suggesting its use should be combined with other relevant clinical techniques in the practice of the specialty, thus allowing for better prognostic outlining as well as the development of new therapeutic schemes to contribute to improvements in survival rates and the quality of life of these patients. Patient stratification is particularly important to prevent patients who will not benefit from certain treatments from undergoing them, thereby avoiding toxicity, allowing time for other treatments to be considered, and saving on the cost of treatments.

To the best of our knowledge, this is the first study evaluating the PD-L1 tumor expression and clinicopathological data of the Brazilian advanced-stage melanoma population, and their association with ICB response, in a real-world scenario. Here, we validated some previously reported biomarkers such as PD-L1 tumor and immune cells’ expression [37], and *BRAF* V600 mutations [38], in a very heterogeneous population. Our results are also in accordance with other studies [10,38] in which patients receiving anti-PD1 monotherapy or a combination of anti-PD1 and anti-CTLA-4 responded better than those patients treated with anti-CTLA-4 alone. Moreover, a recent clinical trial has demonstrated that using ICB as first line is beneficial for advanced melanoma patients [39], and new ICB combinations show greater treatment outcomes [40].

One limitation of this study is the fact that most of the samples for PD-L1 analysis were from the primary tumors and not from the metastatic sites. This is due to the study’s retrospective nature and the difficulty of obtaining samples from visceral organs in which most melanoma metastases are located. Prospective studies are needed to validate our findings.

In conclusion, due to high ICB cost constraints, biomarker-driven selection of advanced melanoma patients should be implemented as a routine practice to predict treatment response as well as overall survival. This would reduce costs and improve the therapeutic efficacy of ICB therapies. 

## Figures and Tables

**Table 1:** Clinicopathological and treatment characteristics of the 210 advanced melanoma patients undergoing immune checkpoint blockade, by patient response groups.

Characteristic	Category	All Patients *n* = 210 (%)	Responders *n* = 83 (%)	Non-Responders *n* = 127 (%)	*p*-Value
Sex	Male	123 (58.6)	47 (56.6)	76 (59.8)	0.669
	Female	87 (41.4)	36 (43.4)	51 (40.2)	
Race	Caucasian	181 (86.2)	71 (85.5)	110 (86.6)	0.827
	Non-Caucasian	24 (11.4)	10 (12.0)	14 (11.0)	
	*Missing*	5 (2.4)	2 (2.4)	3 (2.4)	
Primary tumor site	Lower limbs	66 (31.4)	20 (24.1)	46 (36.2)	0.245
	Trunk	45 (21.4)	23 (27.7)	22 (17.3)	
	Head and neck	39 (18.6)	15 (18.1)	24 (18.9)	
	Upper limbs	22 (10.5)	10 (12.0)	12 (9.4)	
	Other	9 (4.3)	2 (2.4)	7 (5.5)	
	Primary unknown	29 (13.8)	13 (15.7)	16 (12.6)	
Histological subtype	Nodular	38 (18.1)	15 (18.1)	23 (18.1)	**0.020**
	Acral lentiginous	37 (17.6)	11 (13.3)	26 (20.5)	
	Superficial spreading	31 (14.8)	20 (24.1)	11 (8.7)	
	Other	22 (10.5)	7 (8.4)	15 (11.8)	
	*Missing*	82 (39.0)	30 (36.1)	52 (40.9)	
Pathological staging	III	9 (4.3)	2 (2.4)	7 (5.5)	0.249
	IVa	32 (15.2)	10 (12.0)	22 (17.3)	
	IVb	52 (24.8)	26 (31.3)	26 (20.4)	
	IVc	58 (27.6)	20 (24.1)	38 (29.9)	
	IVd	36 (17.1)	12 (14.5)	24 (18.9)	
	*Missing*	23 (11.0)	13 (15.7)	10 (7.9)	
*BRAF* status	V600 mutated	77 (36.7)	38 (45.8)	39 (30.7)	0.132
	Wild Type (WT)	106 (50.5)	40 (48.2)	66 (52.0)	
	*Missing*	27 (12.9)	5 (6.0)	22 (17.3)	
*NRAS* status	Mutated	10 (4.8)	2 (2.4)	8 (6.3)	0.094
	WT	67 (31.9)	34 (41.0)	33 (26.0)	
	*Missing*	134 (63.3)	47 (56.6)	86 (67.7)	
Treatment agent	Nivolumab (anti-PD-1)	116 (55.2)	49 (59.0)	67 (52.8)	**0.021**
	Pembrolizumab (anti-PD-1)	56 (26.7)	24 (28.9)	32 (25.2)	
	Ipilimumab (anti-CTLA-4)	28 (13.3)	4 (4.8)	24 (18.9)	
	Nivolumab + NKTR-214 (IL-2 agonist)	8 (3.8)	4 (4.8)	4 (3.1)	
	Nivolumab + ipilimumab	2 (1.0)	2 (2.4)	0 (0.0)	
Treatment agent grouped	Anti-PD1	172 (81.9)	73 (88.0)	135 (81.8)	**0.009**
	Anti-CTLA-4	28 (13.3)	4 (4.8)	24 (18.9)	
	Anti-PD-1 + IL-2 agonist	8 (3.8)	4 (4.8)	4 (3.1)	
	Anti-PD-1 + anti-CTLA-4	2 (1.0)	2 (2.4)	0 (0.0)	
ICB treatment line	First	113 (53.8)	56 (67.5)	57 (44.9)	**0.001**
	Second	67 (31.9)	23 (27.7)	44 (34.6)	
	Greater than or equal to third	30 (14.3)	4 (4.8)	26 (20.5)	
Treatment response	Disease progression (DP)	127 (60.5)	0 (0.0)	127 (100.0)	**<0.001**
	Stable disease (SD)	34 (16.2)	34 (41.0)	0 (0.0)	
	Partial response (PR)	36 (17.1)	36 (43.4)	0 (0.0)	
	Complete response (CR)	13 (6.2)	13 (15.7)	0 (0.0)	

**Table 2:** Origin of tumor tissue samples from patients.

Source of FFPE Sample	*n* (%)
Primary tumor	113 (53.8)
Subcutaneous metastasis	59 (28.1)
Lymph nodal metastasis	21 (10.0)
Metastasis in other organs	17 (8.1)

**Table 3:** Univariate and multivariate analyses evaluating the effect of clinical and molecular features on the therapeutic response of advanced melanoma patients treated with ICB.

Characteristic	Category	Univariate Analysis *p*-Value	Regression Coefficient (95% CI)	Multivariate Analysis *p*-Value
*BRAF* status	V600 mutated	0.132	Reference	
	Wild Type (WT)		1.297 (0.689–2.444)	0.420
	*Missing* = 27			
Treatment agent grouped	Anti-PD1	**0.009**	Reference	
	Anti-CTLA-4		0.712 (0.179–2.824)	0.629
	Anti-PD-1 + Anti-CTLA-4		0.0 (0.0–0.0)	0.999
	Anti-PD-1 + IL-2 agonist		0.999 (0.227–4.402)	0.999
ICB treatment line	First	**0.001**	Reference	
	Second		1.651 (0.871–3.132)	0.125
	Greater than or equal to third		6.087 (1.970–18.812)	**0.002**
PD-L1 expression	Negative (≤1%)	**0.005**	Reference	
	Positive (>1%)		0.394 (0.193–0.803)	**0.010**

## Data Availability

The data that support the findings of this study are available upon request from the authors.
